# Current Status of Pearl Millet Downy Mildew Prevalence across Agroecological Zones of Senegal

**DOI:** 10.1155/2019/1252653

**Published:** 2019-07-07

**Authors:** Yedomon Ange Bovys Zoclanclounon, Ghislain Kanfany, Aboubacry Kane, Daniel Fonceka, Georgina Lala Ehemba, Fatmata Ly

**Affiliations:** ^1^Centre d'Etudes Régional pour l'Amélioration de l'Adaptation à la Sécheresse, BP 3320, Route de Khombole, Thiès, Senegal; ^2^West Africa Centre for Crop Improvement, University of Ghana, PMB 30, Accra, Ghana; ^3^Centre National de Recherches Agronomiques de Bambey, Institut Sénégalais de Recherches Agricoles, BP 211, Bambey, Senegal; ^4^Faculté des Sciences et Techniques, Université Cheikh Anta Diop de Dakar, BP 5005, Fann, Dakar, Senegal

## Abstract

Pearl millet is a dominant staple cereal crop for smallholder farmers in Senegal. However, the crop is constrained by various nonbiotic and biotic stresses such as downy mildew disease. To assess the prevalence of this disease in Senegal, a field survey was conducted during the rainy season of 2017 across eight main pearl millet production regions following latitudinal gradient with different climatic conditions. Results showed that downy mildew prevalence was higher in Kaolack (incidence = 68.19%), Kaffrine (incidence = 77.19%), Tambacounda (incidence = 97.03%), Sedhiou (incidence = 82.78%), and Kolda (incidence = 98.01%) than Thies (incidence = 28.21%), Diourbel (incidence = 24.46%), and Fatick (incidence = 37.75%) regions. The field survey revealed an incidence as high as 98% and 28% of infected area in surveyed fields. Significant correlations between geographic coordinates, disease incidence, and infected areas were also observed. This study provided information that could help to understand the prevalence of downy mildew in pearl millet in Senegal.

## 1. Introduction

Pearl millet is an important cereal crop in Senegal. It is extensively grown in Diourbel, Fatick, Kaolack, Kaffrine, Tambacounda, and Kolda regions, located in the central, southern, and south-eastern parts of the country. Its production in these regions accounted for 329,494 tons in 2014 growing season which represents 80% of total national production [1]. Pearl millet is used as food crop for human consumption and as fodder for livestock. Its adaptation to drought and low soil fertility makes this plant a resilient model species to adverse effects of climate change [2]. Nutritional composition of pearl millet shows relatively higher level of protein, iron, and zinc compared to rice, sorghum, and maize [3, 4]. Pearl millet is a potential candidate food crop to address nutritional needs and food insecurity occurring in countries with high levels of malnutrition [5, 6]. With the expected increase of temperature (up to 2.6°C) by 2050 [7] and in Senegalese population (40 million) [8], pearl millet is expected to play a crucial role to ensure food security in Senegal.

Fungal, bacterial, and viral pathogens as well as nematodes represent biotic constraints to pearl millet production [9]. However, downy mildew caused by* Sclerospora graminicola* [(Sacc.) J. Schröt.] is considered as the most destructive disease of pearl millet with significant economic impact on grain yield and fodder quality in drought-prone areas in Africa and Asia [10, 11]. A disease incidence of 45-95% and a yield loss of 20-80% have been reported by [12, 13].

In Senegal, downy mildew incidence of 65% and subsequent low yield of 487 kg ha^−1^ were reported in the T99B genotype during the rainy season of 2004 [14]. Recently, Kanfany et al. [15] identified 55 resistant inbred lines and highlighted the existence of pathogenic variation across four agricultural research stations of National Agricultural Institute of Senegal [16]. However, the real status of downy mildew prevalence in rural area conditions is not well known. The holistic overview of the occurrence of this disease in rural area may provide a blueprint for efficient breeding for downy mildew resistance of pearl millet. The present study aimed to determine the prevalence of downy mildew across the main pearl millet production areas in Senegal following longitudinal and latitudinal gradients.

## 2. Materials and Methods

### 2.1. Sampling Plan for Field Survey

**Figure 1:**
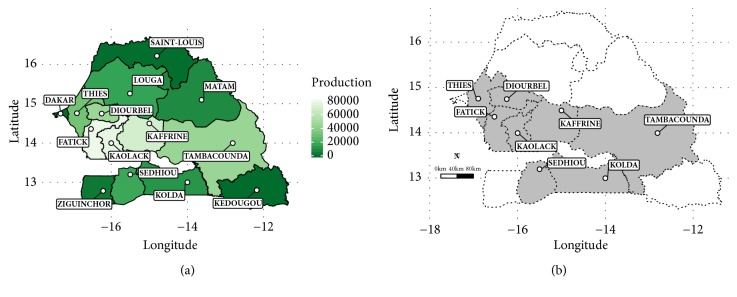
Map showing production (ton) of pearl millet in regions of Senegal during agricultural campaign 2014-2015: (a) gray-colored regions representing on-farm surveyed ones (production ≥ 15000 tons) during rainy season 2017 (b).

Downy mildew survey was conducted in Thies, Diourbel, Fatick, Kaolack, Kaffrine (West Central Agricultural “Peanut Basin” and Saloum Agricultural Regions), Tambacounda (South-Eastern Region), Kolda, and Sedhiou (Casamance) (Figure 1(b)). The most productive regions (≥15,000 tons per year) were selected for this survey (Figure 1(a)).

**Figure 2:**
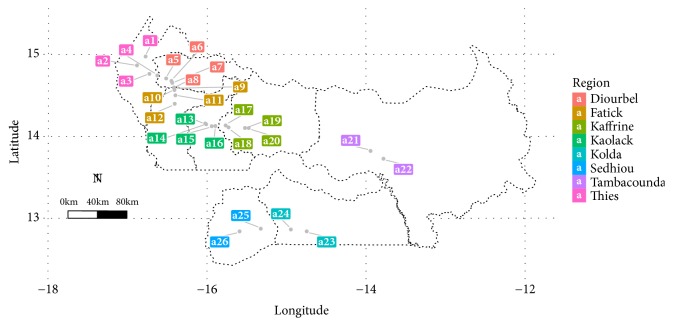
Map showing surveyed fields across the pearl millet productive regions of Senegal during rainy season 2017.

The sampling sites were selected based on latitudinal gradient and, then, 26 fields (Figure 2) were visited.

Rainfall, relative humidity, and temperatures data were provided by the regional weather channels of the National Agency of Civil Aviation and Meteorology of Senegal. In each region, diseased pearl millet plants were sampled in the fields at every 10 km along roadside. Each sampling was made by using a stratified random sample design by Delp et al. [17]. A square survey area of 60 m × 60 m was delimited and then divided into nine plots of 20 m × 20 m. Typical symptoms (Figure 5) of downy mildew were checked before the setting up of the observational plots.

Disease incidence was calculated as follows:

(1) Disease  incidence%=Number  of  plants  showing  the  symptomsTotal  number  of  sampled  plants×100

Downy mildew infected areas (m^2^) per surveyed field were determined using area calculation function of Garmin® GPS.

### 2.2. Data Analysis

GPS coordinates and downy mildew incidence data were recorded. Mapping representation was used to visualize downy mildew incidence and infected area data. A proportional symbol map represented by spatial point data with circle symbol was projected on Senegalese boundaries map according to a mathematical scaling methodology defined by Tanimura* et al*. [18] as follows:

(2) $$\begin{array}{c} r_{i}=\sqrt{\left(\frac{V_{i}}{V_{max}}\right)}\times r_{max} \end{array}$$

where r_i_ is the radius of the circle to be drawn; r_max_ is radius of the largest circle on the map; v_i_ is value of downy mildew data variable for which the circle was drawn, and v_max_ is the maximum value of downy mildew data variables.

The data visualization maps were realized using ggplot2 package [19]. To investigate the dependence between climatic, geographic parameters, and downy mildew variables, Pearson's correlation test was computed in R using ggcorr function of the package GGally [20]. All maps and statistical analysis were performed using R (version 3.4.5) statistical and visualization data analysis software [21].

## 3. Results

### 3.1. Temperature, Relative Humidity, and Rainfall Variability

**Figure 3:**
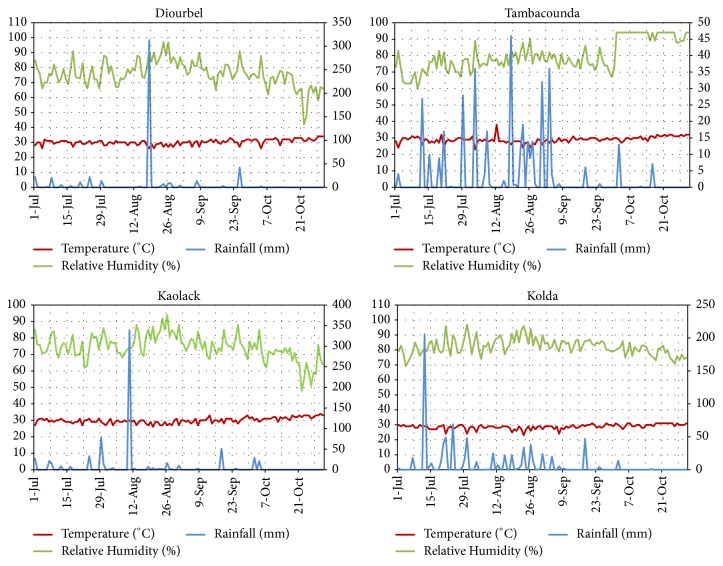
Daily temperature, relative humidity (both at left-side of the Y-axis), and rainfall (right-side of the Y-axis) in Diourbel, Tambacounda, Kolda, and Kaolack during rainy season 2017.

**Figure 4:**
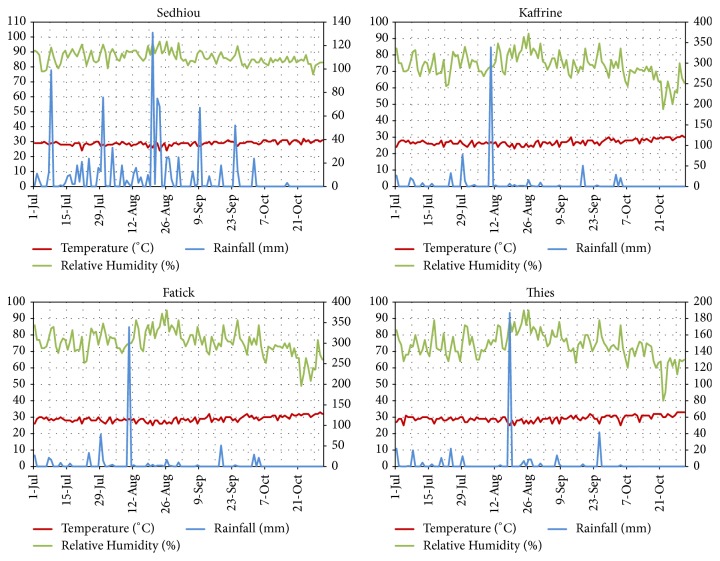
Daily temperature, relative humidity (both at left-side of the Y-axis), and rainfall (right-side of the Y-axis) in Sedhiou, Thies, Kaffrine, and Fatick during rainy season 2017.

**Figure 5:**
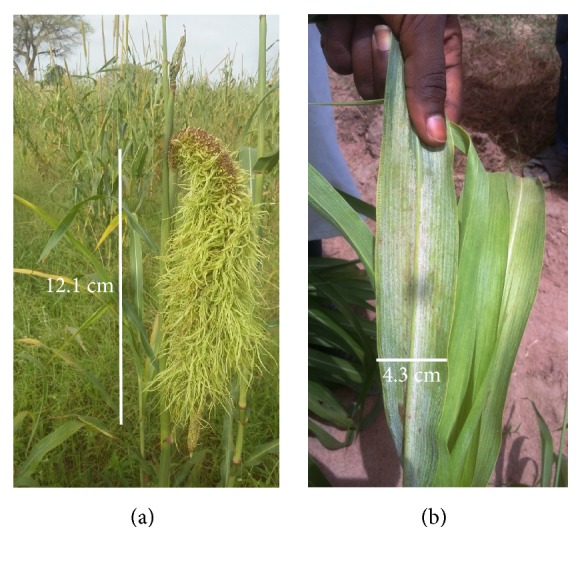
Typical downy mildew symptoms: panicle malformation (a) and abaxial sporulation on leaf (b) observed during on-farm downy mildew survey.

The weather conditions in each region are presented in Figures 3 and 4. Daily temperatures in Kolda ranged from 23 to 31°C with the averages of 82% relative humidity.

Similar trends of temperature were registered in Tambacounda (28.98±1.96°C), Kaolack (75.95±8.60°C), and Diourbel (30.25±7.16°C). The maximum rainfall, 206 mm, occurred at Kolda in July. However, Tambacounda presented regular rainfalls from July 12th to September 7th corresponding to a cumulative value of 340.86 mm.

### 3.2. On-Farm Downy Mildew Prevalence

This study revealed the occurrence of downy mildew in all surveyed regions. Downy mildew incidence recorded across the different fields ranged from 8.9 to 98.09% (Table 1).

**Figure 6:**
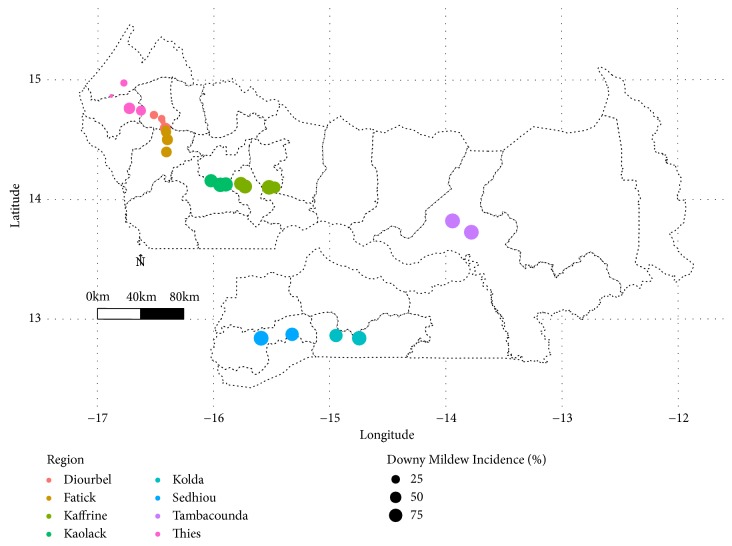
Map showing downy mildew incidence across surveyed field in Senegal during rainy season 2017.

**Figure 7:**
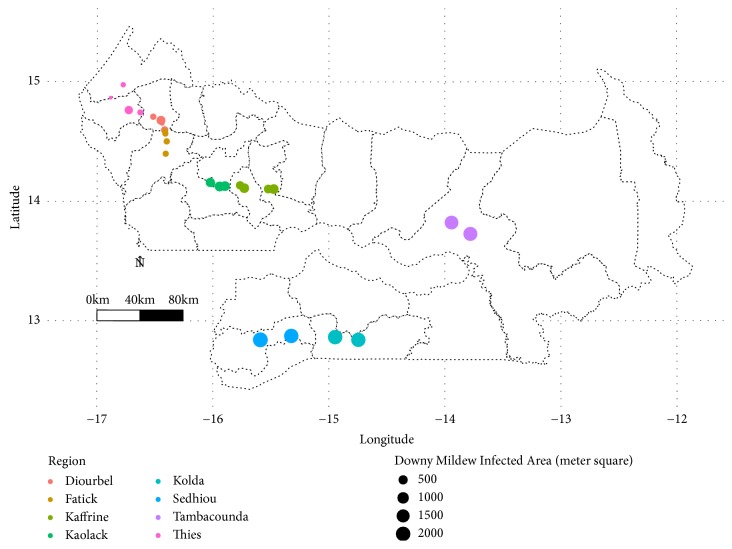
Map showing infected area across surveyed field in Senegal during rainy season 2017.

Fields located in Sedhiou region had the highest incidence with an infected area of 4,138.49 m^2^ among 14,085 m^2^ surveyed. Downy mildew incidence and infected area increased from latitude 15.00° to 13.00° (Figures 6 and 7).

Following longitudinal gradient, high values of downy mildew incidence (≥ 75%) and infected area (≥ 1000 m^2^) were recorded between longitudes -16.00° and -14.00° (Figures 6 and 7).

Downy mildew hotspot zones were located between longitudes -16° and -13.5° and latitudes 12.5°-14.5° (Figures 6 and 7). These hotspot zones were located in Fatick, Kaolack, Kaffrine, Tambacounda, Kolda, and Sedhiou regions. On-farm survey revealed that two pearl millet cultivars were widely grown across surveyed regions. Souna cultivar was the main pearl millet cultivar grown in Thies, Diourbel, Fatick, Kaolack, Kaffrine, and Tambacounda while Sanio cultivar was extensively planted in Sedhiou and Kolda regions. The cultivar Souna, the same as in Sanio, has shown susceptibility to the disease (Table 2). However, Sanio cultivar has a higher downy mildew incidence (85%) compared to Souna (51%).

### 3.3. Relationship between Downy Mildew and Climatic and Geographical Parameters

**Figure 8:**
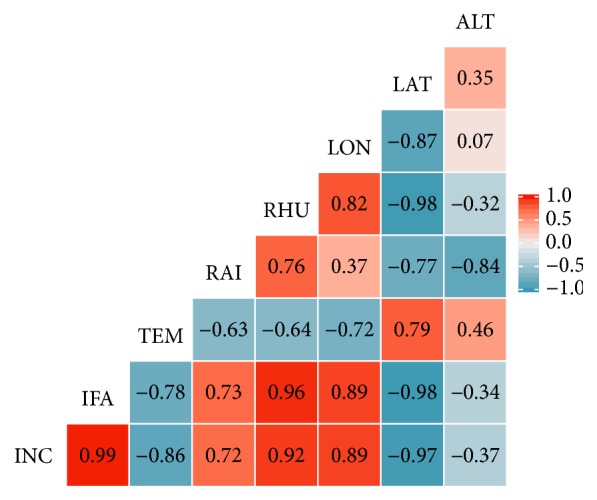
Correlation between climatic, geographic, and downy mildew incidence. INC: incidence, IFA: infected area, TEM: temperature, RAI: rainfall, RHU: relative humidity, LON: longitude, LAT: latitude, and ALT: altitude.

Correlation analysis showed significant dependence of downy mildew incidence with latitudinal gradient (r = -0.76). Thus, increase in latitudinal gradient resulted in a significant decrease of downy mildew incidence. Similar results were also observed with a negative correlation (r = -0.84) between latitudinal gradient and infected area by downy mildew (Figure 8).

Pearson's correlation test results revealed a positive correlation (r = 0.76) between longitudinal and downy mildew incidences at 0.05 levels of significance. Longitude gradient also positively correlated to downy mildew infected area (r = 0.76; p < 0.05). Among climatic variables, relative humidity strongly correlated (r = 0.82; p < 0.05) to infected area by downy mildew. Downy mildew incidence and infected area were highly correlated (r = 0.92; p < 0.01) (Figure 8).

## 4. Discussion

This study provided information about downy mildew prevalence in pearl millet production areas of Senegal. The presence of downy mildew in Senegal has also been reported by Girard [22] and recently confirmed by Kanfany et al. [16] in the major pearl millet production regions in Senegal. This highlights the wide distribution of the disease which is known to be present in the major pearl millet-growing regions. The prevalence of the disease increased as the latitudinal gradient decreased and hotspot areas were also highlighted. High rainfall and relative humidity observed at low latitudes (Sedhiou and Kolda regions) could explain this result. Downy mildew was also highly present at higher latitudes (Kaolack and Kaffrine regions). Indeed, most of pearl millet production in Senegal was mainly provided from these regions which could increase infestation rate by* Sclerospora graminicola*. Results from this study revealed lower incidences and infected areas in Thies, Diourbel, and Fatick regions compared to Kaolack and Kaffrine regions. This suggests virulence variation in* S. graminicola *across surveyed zones. These results were confirmed by Kanfany et al. [16]. The presence of genetic variability may also be suspected.

Results showed that relative humidity had a significant effect in downy mildew distribution. Shetty* et al*. [23] reported that relatively high temperature up to 30°C and relative humidity (80-95%) are key conditions for* S. graminicola *development and propagation. This could explain the high prevalence of downy mildew during the rainy season. Other parameters such as wind [11] and local cultural practices can also promote the survival and spread of* S. graminicola*. Some farmers use the same field area for pearl millet cultivation over years resulting in potentially high level accumulation of the pathogen inoculum in the soil. Furthermore, only two cultivars (Souna and Sanio) are preferentially sown every year. This absence of variability of the genetic resources may be detrimental in case of highly virulent host-specific pathotypes advent.

This study showed that Kaolack, Fatick, Tambacounda, Sedhiou, and Kolda regions had the highest infected areas in Senegal. Thus, downy mildew disease could represent a major constraint for small scale farmers in their efforts to achieve food security. Pathogenic, genetic variability and management means of isolates collected from surveyed fields should be investigated as key points for further study.

## Figures and Tables

**Table 1:** Prevalence of downy mildew across surveyed regions of Senegal following longitude and latitude gradient during rainy season 2017.

Region	Field surveyed		Area surveyed (m^2^)		DM Incidence (%)	
	Total	Infected	Total	Infected	Mean	Min-Max
Thies	4	4	66300	473.50	28.21	8.90-50.95
Diourbel	4	4	62436	789.02	24.46	10.86-45.98
Fatick	4	4	41739	356.40	37.75	22.10-46.97
Kaolack	4	4	131656	1926.71	68.19	20.07-91.84
Kaffrine	4	4	90978	1963.83	77.19	55.85-92.87
Tambacounda	2	2	25828	3599.48	97.03	95.96-98.09
Sedhiou	2	2	14085	4138.49	82.78	76.02-96.97
Kolda	2	2	24547	3957.97	98.01	74.46-91.10

Total	26	26 (100%)	457569	17205.44	56.76	8.9-98.09

**Table 2:** Prevalence of downy mildew on cultivars across pearl millet production regions.

Cultivar	Field surveyed		Area surveyed (m^2^)		DM Incidence (%)	
	Total	Infected	Total	Infected	Mean	Min-Max
Souna	22	22	418937	9108.94	51.90	8.90-98.09
Sanio	4	4	38632	8096.46	84.64	47.87-96.97

Total	26	26 (100%)	457569	17205.44	68.27	8.90-98.09

## Data Availability

The data used to support the findings of this study are included within the supplementary information file.

## Supplementary Materials

Supplementary Materials The data are on excel file and are located on sheet one. In the excel file you will find geographical and downy mildew incidence information.
